# Differences in COVID-19 Vaccine Uptake Among Immigrants and Non-Immigrants in the United States: Evidence from MEPS

**DOI:** 10.1093/geroni/igaf122.803

**Published:** 2025-12-31

**Authors:** Mohammad Usama Toseef, Indrakshi Roy, Nasim Ferdows

**Affiliations:** Corewell Health, Farmington Hills, Michigan, United States; Indiana University, Bloomington, Bloomington, Indiana, United States; Northeastern University, Boston, Massachusetts, United States

## Abstract

**Background:**

Understanding differences in COVID-19 vaccine uptake is crucial for addressing public health inequities. Immigrants in the United States face unique structural and socioeconomic barriers that may influence vaccination rates, including access to healthcare, acculturation, and trust in medical institutions. This study examines differences in COVID-19 vaccine uptake among immigrant and non-immigrant populations, with attention to the role of socioeconomic and demographic factors.

**Methods:**

This study utilizes data from the 2021-2022 Medical Expenditure Panel Survey (MEPS), a nationally representative survey of the U.S. non-institutionalized population. The analytic sample includes 39,767 adults aged 18 and older. Immigration status is categorized as U.S.-born, foreign-born residing in the U.S. for less than 15 years, and acculturated immigrants (residing in the U.S. for 15 years or more). Logistic regression models, adjusted for sociodemographic factors, estimate the association between immigration status and COVID-19 vaccine uptake.

**Results:**

Preliminary unweighted analyses indicate higher COVID-19 vaccination rates among foreign-born individuals compared to U.S.-born populations. However, differences emerge when considering acculturation, with acculturated immigrants showing a distinct vaccination trajectory. Adjusted models will further explore the impact of socioeconomic factors, such as education and income, on vaccination likelihood.

**Conclusion:**

Findings suggest that immigration status and acculturation play significant roles in shaping COVID-19 vaccine uptake. Future research should explore barriers to vaccination among acculturated immigrants and identify targeted interventions to improve vaccine accessibility and trust among immigrant communities.

